# Network Pharmacology Based Research on the Combination Mechanism Between Escin and Low Dose Glucocorticoids in Anti-rheumatoid Arthritis

**DOI:** 10.3389/fphar.2019.00280

**Published:** 2019-03-22

**Authors:** Leiming Zhang, Yanan Huang, Chuanhong Wu, Yuan Du, Peng Li, Meiling Wang, Xinlin Wang, Yanfang Wang, Yanfei Hao, Tian Wang, Baofeng Fan, Zhuye Gao, Fenghua Fu

**Affiliations:** ^1^Key Laboratory of Molecular Pharmacology and Drug Evaluation, Ministry of Education, School of Pharmacy, Yantai University, Yantai, China; ^2^The Biomedical Sciences Institute of Qingdao University (Qingdao Branch of SJTU Bio-X Institutes), Qingdao University, Qingdao, China; ^3^College of Arts and Sciences, Shanxi Agricultural University, Taigu, China; ^4^Air Force General Hospital, PLA, Beijing, China; ^5^Xiyuan Hospital, China Academy of Chinese Medical Sciences, Beijing, China

**Keywords:** rheumatoid arthritis, glucocorticoids, glucocorticoid receptor, Escin, dexamethasone

## Abstract

Rheumatoid arthritis (RA) is characterized by chronic progressive symmetrical synovitis and destruction of multiple joints. Glucocorticoids (GCs) are widely used in the treatment of RA. However, their adverse effects can be serious. Escin, which is isolated from *Aesculus hippocastanum* L., has been reported to have anti-inflammatory effects. We investigated the anti-RA effect of Escin combined with low dose GCs (dexamethasone, Dex) and the underlying mechanism. Adjuvant-induced RA rats and lipopolysaccharides (LPS)-injured RAW264.7 cells were used to investigate the anti-RA effects of Escin combined with low dose Dex *in vivo* and *in vitro*. The results showed that Escin combined with low-dose Dex significantly decreased arthritic index, serum IL-6 and TNF-α levels, reduced paw swelling, and ameliorated the joint pathology and immune organ pathology. Gene chip results revealed that *Nr3c1* (GR) expression was significantly altered, and that GR was activated by Escin and low dose Dex *in vivo* and *in vitro*. Additionally, Escin combined with low dose Dex also significantly increased GR mRNA expression. However, when GR expression was suppressed by its specific inhibitor, the anti-RA effect of Escin combined with low-dose Dex was abolished. The data in this study demonstrated that Escin combined with Dex reduced the dose of Dex, and exerted significant anti-RA effects, which could also reduce the adverse effects of Dex. This combination might result from GR activation. This study might provide a new combination of drugs for the treatment of RA.

## Introduction

Rheumatoid arthritis (RA) is a chronic autoimmune disease that causes progressive articular damage, functional loss, and comorbidity. RA affects about 1% of the population, and it can present at any age (McInnes and Schett, 2017). Its physiopathology includes synovial inflammation with pro-inflammatory cytokine overexpression (Courbon et al., 2018).

Glucocorticoids (GCs) are widely used in the treatment of RA. However, long-term and high dose GC use can lead to serious adverse effects, such as immunosuppression, osteoporosis and metabolic disorders (Gossye et al., 2009). One of the methods to minimize the undesirable adverse effects is through administering them with other pharmaceuticals, aiming at synergistic effects to reduce the dose and duration of cortical therapy.

Escin (polyhydroxyolean-12-ene 3-*O*-monodesmosides) is a natural mixture of triterpene saponins isolated from *Aesculus hippocastanum* L. (Xin et al., 2011a). Several studies have reported that it has anti-inflammatory (Wang et al., 2013), anti-edematous (Wang et al., 2011), and anti-cancer properties (Cheong et al., 2018). It was also reported that Escin could synergize with GCs to enhance their anti-inflammatory effect (Xin et al., 2011b).

Recent years, network-based approaches offer the potential to explore, in a systematic way (Hopkins, 2008), the effect of a drug candidate in a global physiological environment (Xiong et al., 2019), and was commonly used to decipher new drug-target relationships in drug discovery (Boezio et al., 2017). And this method was also used in this study to give a constructive result of the Escin effect on RA. Based on the network pharmacology, we investigated whether the combination of Escin and GCs would improve the anti-RA effects and the underlying mechanism.

## Materials and Methods

### Materials

Escin, including sodium aescinate tablets (batch no: 201509321) and sodium aescinate for injection (batch no: 2017110103), were purchased from Shandong Luye Pharmaceutical Co., Ltd. (Yantai, China). Dexamethasone tablets (Dex, batch no: 160601201) were purchased from Chen Xin Pharmaceutical Co., Ltd. (Jining, China). Dexamethasone powder (D4902, purity ≥ 97%), lipopolysaccharides (LPS) and 3-(4,5-dimethyl-2-thiazolyl)-2,5-diphenyl-2-H-tetrazolium bromide (MTT) were purchased from Sigma-Aldrich (St. Louis, MO, United States). Complete Freund’s adjuvant (CFA) (containing 10 mg/ml of dry, heat-killed Mycobacterium tuberculosis) was purchased from Chondrex Co., Ltd. (Redmond, WA, United States, batch no: 170334). RPMI medium 1640 was purchased from Gibco (Carlsbad, CA, United States, batch no: 1960297). Fetal bovine serum (FBS) was purchase from Zhejiang Tianhang Biological Technology Co., Ltd. (Hangzhou, China, batch no: 11011-8611). Griess reagent kit (S0023) was purchased from Beyotime Institute of Biotechnology (Haimen, China). Primary antibody for IκBα (K2017), and GR (A2518) were purchased from Santa Cruz Biotechnology (Santa Cruz, CA, United States). Primary antibody for *p*-IκBα (S32) and *p*-p65 (S536) were purchased from Cell signaling Technology (Danvers, MA, United States). P65 (ab16502) was purchased from Abcam company (Burlingame, CA, United States). Second antibodies for anti-rabbit IgG HRP-linked antibody and anti-mouse IgG HRP-linked antibody were purchased from Beyotime Institute of Biotechnology (Haimen, China). RU486 (m8046) were purchased from Sigma-Aldrich (United States). qPCR reagents: UltraSYBR Mixture (CW2602M) were purchased from Kang Wei century Biotechnology Co., Ltd. (Beijing, China).

### Animals

Animal experimental procedures were performed in strict accordance with the National Institutes of Health Regulations on the use and care of animals for scientific purposes. Male Sprague-Dawley rats (weight, 180–220 g) were purchased from Shandong Jinan PengYue Experimental Animal Breeding Co., Ltd. (Jinan, China). All the rats were housed in diurnal lighting conditions (12 h/12 h) and allowed free access to food and water. All experimental procedures in this study were performed in accordance with the Guidelines for the Care and Use of Laboratory Animals of Yantai University, and were approved by the ethics committee.

### Induction of Adjuvant-Induced Rheumatoid Arthritis

Adjuvant-induced RA (AIA) was induced in the rats according to the method described by Pearson (Pearson, 1963). Briefly, the animals were inoculated with a subplantar injection of 0.1 mL CFA into the right hind paw at day 0 and were randomly allocated to 6 groups of eight rats each (*n* = 10) as follows: the control group; AIA group; AIA + Dex (0.2 mg ⋅ kg^−1^) group; AIA + Dex (0.05 mg ⋅ kg^−1^) group; AIA + Escin (10 mg ⋅ kg^−1^) group; AIA + Escin (10 mg ⋅ kg^−1^) + Dex (0.05 mg ⋅ kg^−1^) group. Dex and Escin were administered orally 15 days after inoculation, and once daily for a period of 2 weeks (from day 15 to day 28). Body weight was determined on Day 0, 3, 6, 9, 12, 15, 18, 21, 24, and 27.

### Measurement of Paw Volume

Paw volume was measured using a Plethysmometer (YLS-7B, Shandong Academy of Medical Sciences, Jinan, China) on Day 0 before CFA injections and thereafter on Day 3, 7, 11, 15, 18, 22, 25, and 28. The change in paw volume was calculated as the difference between the final (28 days) and initial (0 day) paw volume (Lee et al., 2009).

### Measurement of Arthritic Index

The morphological features of arthritis such as redness, swelling and erythema were determined using a five-point ordinal scale (0–4) scoring system as follows: 0, narrow paw; 1, mild swelling and erythema of the digits; 2, swelling and erythema of the digits; 3, severe swelling and erythema; 4, gross deformity and inability to use the limb (Paval et al., 2009). Thus, the maximum score for both the paws was 8.

### Histopathological Examination

After 2 weeks of Dex/Escin administration, the speen, thymus, right leg joint, and left leg joint tissues with pathologic changes were stained with haematoxylin-eosin (HE) to evaluate the effect of Dex and Escin. The tissues were removed and fixed in 10% buffered formalin (pH = 7.0) for 3 days. Samples were then trimmed, decalcified in 10% EDTA at room temperature for 60 days, embedded in paraffin and cut into slices. Sections (4 μm) were stained with HE. The histopathological changes and severity were observed with a light microscope (Pan et al., 2017).

### Immune Organ Index

After Dex/Escin administration for 2 weeks, the spleen and thymus weights were determined and the organ-to-body weight ratio was calculated.

### Glucocorticoid Signaling RT2 Profiler OCR Array

The GC signaling gene changes (including 84 genes) in synovial tissue of the joints were detected by Shanghai Bo Hao Biotechnology Co., Ltd. (Shanghai, China) using a RT2 Profiler PCR Array. Briefly, the procedure started with conversion of experimental RNA samples into first-strand cDNA using the RT2 First Strand Kit. Then, the cDNA was mixed with an appropriate RT2 SYBR Green Mastermix. This mixture was aliquoted into the wells of the RT2 Profiler PCR Array. PCR was performed and the relative expression was determined using data from the real-time cycler and the ΔΔCT method.

### Cell Culture

Murine RAW264.7 macrophages obtained from the American Type Culture Collection (ATCC, VA, United States), were cultured in complete RPMI 1640 containing 10% heated-inactivated FBS, 100 U/ml penicillin, and 100 μg/ml streptomycin. Cells were incubated at 37°C in a humidified atmosphere of 5% CO_2_ in air.

### MTT Assay

RAW264.7 cells (5 × 10^4^ cells/well) were cultured in 96-well plates for 24 h after Dex/Escin treatment with or without LPS (1 μg/mL). MTT solution (10 μL; 5 mg/ml) was added, and the cells were incubated at 37°C for an additional 4 h. After washing out the supernatant, the insoluble formazan product was dissolved in DMSO. Then, the optical density was measured at 570 nm using a microplate reader.

### CCK-8 Assay

RAW264.7 cells (5 × 10^3^ cells/well) were cultured in 96-well plates for 24 h after Dex/Escin treatment with or without LPS (1 μg/ml). CCK-8 solution (10 μL) was added to each well of the plate, and the cells were then incubated for 2 h in an incubator. The absorbance was measured at 450 nm using a 96-well plate reader.

### Determination of Nitrite

RAW264.7 cells (1 × 10^5^ cells/well) were cultured in 96-well plates with Dex/Escin pretreatment for 2 h and incubated with LPS for 24 h. Supernatant (50 μl) was collected and mixed with equal volumes of Griess reagent for 10 min at room temperature. Optical density was measured at 540 nm (Wu et al., 2015b).

### Determination of IL-6 and TNF-α

RAW264.7 cells (1 × 10^5^ cells/well) were cultured in 96-well plates with Dex/Escin pretreatment for 2 h and then incubated with LPS for 6 h. The concentrations of IL-6 and TNF-α in the culture medium were determined using ELISA kits, in accordance with the manufactures’ instructions.

After 2 weeks of Dex/Escin administration, rat serum was obtained. The IL-6 and TNF-α serum concentrations were determined using ELISA kits in accordance with the manufactures’ instructions.

### RT-PCR Assay

RAW264.7 cells were seeded in six-well plates treated with Dex/Escin and 1 μg/ml LPS for 30 min. Total RNA was isolated and the RNA concentration was detected using a spectrophotometer. Total RNA (1 μg) was converted to cDNA and real-time PCR (RT-PCR) was performed using a PrimeScript^TM^ RT reagent kit. The PCR primers were as follows: GR, sense 5′-GTTGCGCAGCCTGAATGGCG-3′, antisense 5′-GCCGTCACTCCAACGCAGCA-3′; GAPDH, sense 5′-CGACTTCAACAGCAACTCCCACTCTTCC-3′, antisense 5′-TGGGTGGTCCAGGGTTTCTTACTCCTT-3′. The amplification sequence protocol was conducted using three steps: 1 step: Cycle 1, 95°C 10 min; 2 step: Cycle 40, 95°C 15 s, 60°C 60 s; 3 step: Cycle 1, 95°C 15 s, 60°C 60 s, 95°C 15 s, 60°C 15 s.

### Western Blotting

The expression of *p*-p65, p65, *p*-IκBα, IκBα, and GR in rat tissue and RAW264.7 cells was determined by western blot. The rat lysate and RAW264.7 lysates were incubated on ice for 30 min and then centrifuged at 12,000 rpm for 20 min at 4°C. The supernatant was collected, and the protein concentrations contained in the supernatant were measured using the BCA assay. The protein samples with loading buffer added were boiled for 5 min before loading onto SDS-polyacrylamide gel. After electrophoresis, the gel was electroblotted onto PVDF membranes. Membranes were blocked in Tris-buffered saline (TBS) with 1% Tween-20 (TBST) and 5% non-fat dry milk, and incubated with primary antibody overnight at 4°C. Then, the membranes were washed several times with TBST before incubation with horseradish peroxidase-conjugated secondary antibody for 60 min at room temperature. After subsequent washes in TBST, the protein bands were visualized using the ECL detection kit. The relative intensities of the bands were quantified by densitometric analysis. The densitometric plots of the results were normalized to the intensity of the actin band (Wu et al., 2015a).

### Statistics

Data were expressed as the mean ± standard deviation (SD) of at least three independent experiments. The protective effects were assessed using the GraphPad Prism 6 software (GraphPad, San Diego, CA, United States). Relative protein semi-quantification was performed using the QUANTITY ONE software (Bio-Rad, Hercules, CA, United States). Differences between groups were assessed by ANOVA. A *P*-value less 0.05 was considered statistically significant.

## Results

### Symptom Network of Escin Constructed Using SymMap

**FIGURE 1:**
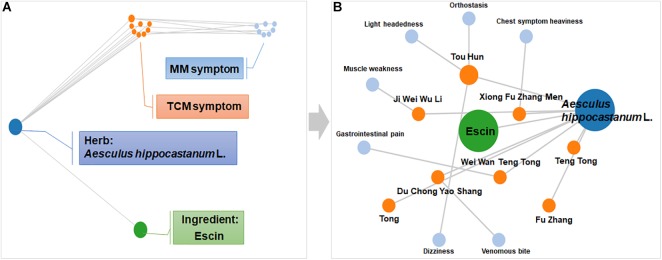
Symptom network of Escin constructed with SymMap. **(A)** Description of network composition. **(B)** The detailed component of Escin network. This network was formed using SymMap (http://www.symmap.org).

Symptom network of Escin was constructed with SymMap^1^ (Wu et al., 2019) to predict the disease that Escin might be intervened. As displayed in Figure 1A, this network including Herb, Ingredient, Traditional Chinese Medicine symptom (TCM symptom), Modern Medicine symptom (MM symptom). The detailed component of Escin network was displayed in Figure 1B. Escin was isolated from *Aesculus hippocastanum* L. TCM symptom related to *Aesculus hippocastanum* L. were Teng Tong, Tong, Du Chong Yao Shang, Ji Wei Wu Li, Xiong Fu Zhang Men, Tou Hun and Wei Wan Teng Tong. Meanwhile, MM symptom correspondent to TCM symptom were Chest symptom heaviness (Xiong Fu Zhang Men), Light headedness, Orthostasis and Dizziness (Tou Hun), Muscle weakness (Ji Wei Wu Li), Gastrointestinal pain (Wei Wan Teng Tong), Venomous bite (Du Chong Yao Shang). Among the symptoms, muscle weakness also occurred in RA. In addition, TCM symptom, Tong, Teng Tong had no related MM symptom until now. However, these symptoms also occurred in RA. This network provide a trace for Escin in anti-RA.

### Effect of Escin Combined With Dex on Arthritic Index and Serum Inflammatory Factor Secretion in AIA Rats

**FIGURE 2:**
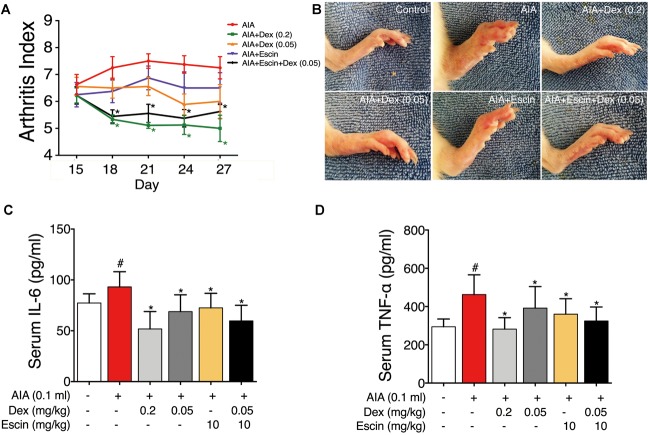
Effect of Escin combined with Dex on arthritic index and serum inflammatory factor secretion in AIA rats. **(A)** Arthritic index was evaluated by 5-point ordinal scale (0–4) scoring system. **(B)** Images for the paws were obtained. **(C,D)** Serum IL-6 and TNF-α were determined with ELISA kits. Data were expressed as the mean ± SD, and were analyzed by ANOVA. ^#^*P* < 0.05 *versus* the control group; ^∗^*P* < 0.05 *versus* the AIA-treated group; Dex, dexamethasone.

The rats gradually developed multiple-joint RA beginning on day 15 after CFA injection. The arthritic index peaked on day 21 (Figure 2A). Dex at daily dose of 0.2 mg/kg and Escin (10 mg/kg) combined with Dex (0.05 mg/kg) significantly reduced the arthritic index compared with the AIA-treated group between days 18 and 27 post-CFA (*P* < 0.05). As illustrated in the representative day 28 paw graphs in Figure 1B, the AIA-treated rats displayed a swelling paw compared with the control rats. After administration of Dex (0.2 mg/kg), Dex (0.05 mg/kg), Escin (10 mg/kg) or Escin (10 mg/kg) combined with Dex (0.05 mg/kg), this swelling was ameliorated (Figure 2B). Moreover, the serum TNF-α and IL-6 concentrations in AIA rats were significantly higher compared with the control group (*P* < 0.05). The serum TNF-α and IL-6 concentrations in the Dex (0.2 mg/kg), Dex (0.05 mg/kg), Escin (10 mg/kg) and Escin (10 mg/kg) combined with Dex (0.05 mg/kg) groups were significantly lower compared with that the AIA-treated group (Figure 2C,D; *P* < 0.05).

### Effect of Escin Combined With Dex on Histopathological Changes and Paw Swelling in AIA Rats

**FIGURE 3:**
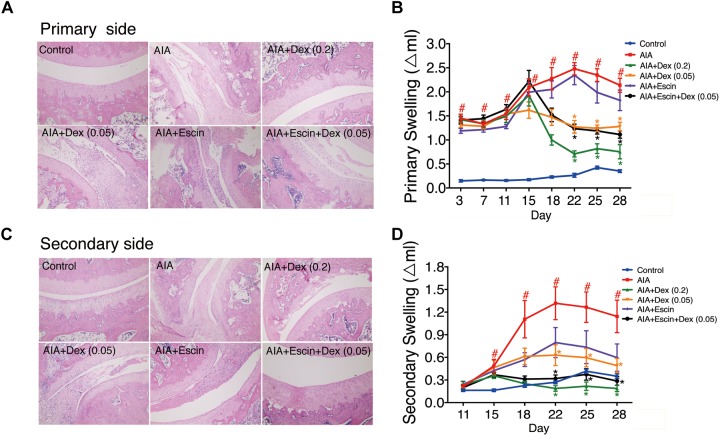
Effect of Escin combined with Dex on histopathological changes and paw swelling in AIA rats. **(A,C)** Histopathological changes of joints in primary side and secondary side were determined with HE staining. **(B,D)** Paw swelling in primary side and secondary side were determined by a Plethysmometer. Data were expressed as the mean ± SD, and were analyzed by ANOVA. ^#^*P* < 0.05 *versus* the control group; ^∗^*P* < 0.05 *versus* the AIA-treated group; Dex, dexamethasone.

Haematoxylin-eosin staining was used to evaluate inflammation and bone lesion induced by AIA. As illustrated in Figure 3, no pathological findings of arthritis were observed in normal joints in the control group. However, the AIA-treated group exhibited severe synovitis, with synovial hyperplasia, inflammatory cells infiltration into the joint cavity, and bone and cartilage erosion on the primary and secondary sides. Treatment with Dex (0.2 mg/kg), Escin (10 mg/kg) combined with Dex (0.05 mg/kg), significantly decreased synovial hyperplasia, cartilage surface erosion, and joints degradation (*P* < 0.05), and substantially reduced the amount of infiltrated inflammatory cells. However, low dose Dex (0.05 mg/kg) and Escin (10 mg/kg) alone did not reverse these effects. After CFA injection, paw swelling on the primary side significantly increased on day 11 and peaked on day 22 (*P* < 0.05). while paw swelling occurred on the secondary side on day 15 and peaked on day 22. Dex (0.2 mg/kg) and Escin (10 mg/kg) combined with Dex (0.05 mg/kg) significantly decreased paw swelling on the primary and secondary sides beginning on day 22 (*P* < 0.05), and the effect of the combined medication was better than that of low-dose Dex (0.05 mg/kg).

### Effect of Escin Combined With Dex on Immune Organ Histopathological Change and Index in AIA Rats

**FIGURE 4:**
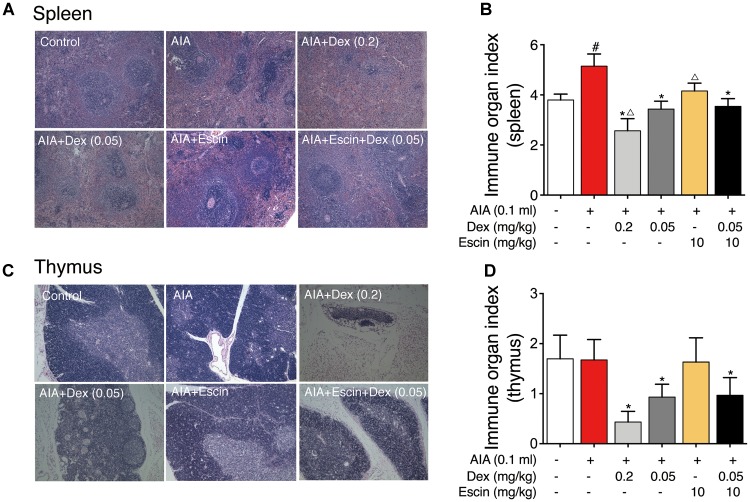
Effect of Escin combined with Dex on immune organ histopathological change and index in AIA rats. **(A,C)** Histopathological changes of spleen and thymus were determined with HE staining. **(B,D)** Immune organ index of spleen and thymus were determined. Data were expressed as the mean ± SD, and were analyzed by ANOVA. ^#^*P* < 0.05 *versus* the control group; ^∗^*P* < 0.05 *versus* the AIA-treated group; ^△^*P* < 0.05 *versus* the Escin (10 mg/kg) combined with Dex (0.05 mg/kg); Dex, dexamethasone.

Red and white pulps were neatly distributed in the spleen of control group. However, in the AIA-treated group, hyperplasia of the white pulp and a larger lymphocyte aggregation area were observed. In the Dex (0.2 mg/kg) group, lymphoid nodules were significantly reduced and atrophied. However, Escin (10 mg/kg) and Escin (10 mg/kg) combined with Dex (0.05 mg/kg) treated groups showed no significant pathological changes (Figure 4A).

Rat thymus tissue from the control and AIA-treated groups showed clear demarcation of the dermal medulla and abundant lymphocytes. In the Dex (0.2 mg/kg) group, the rat thymus tissue medullary volume was atrophied, but the Escin (10 mg/kg) and Escin (10 mg/kg) combined with Dex (0.05 mg/kg) groups showed no significant pathological changes (Figure 4C).

The AIA-treated group displayed a remarkable increase in spleen index compared with the control rats. However, Dex (0.2 mg/kg) caused a marked decrease in spleen index compared with the AIA-treated rats and control rats. Escin (10 mg/kg) and Escin (10 mg/kg) combined with Dex (0.05 mg/kg) showed no marked decrease, compared with Dex (0.2 mg/kg) (Figure 4B). The Dex (0.2 mg/kg) group showed a remarkable decrease in thymus index compared with the AIA-treated rats and control rats (*P* < 0.05). However, Escin (10 mg/kg) and Escin (10 mg/kg) combined with Dex (0.05 mg/kg) showed no marked decrease in thymus index, compared with Dex (0.2 mg/kg) (Figure 4D).

### Pathways Regulated by Escin Combined With Dex in AIA Rats

**FIGURE 5:**
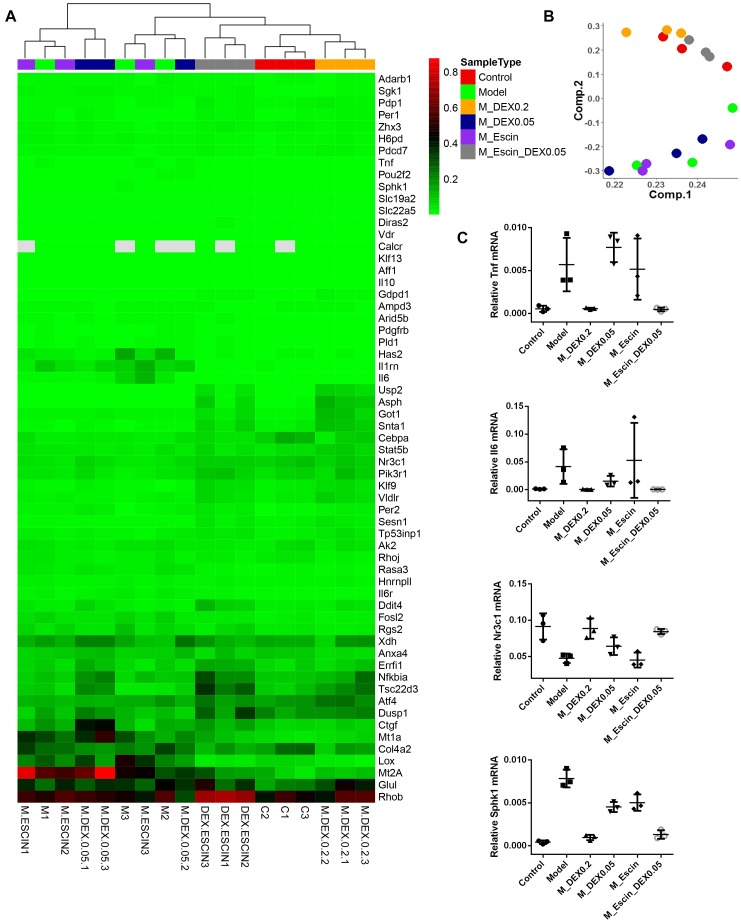
RT^2^ profile PCR array rat glucocorticoid signaling. **(A)** Heatmap analysis of the PCR array. Out of all 84 genes in the array, the heatmap includes 61 genes that were significantly changed in Model *versus* Control, M_DEX0.2 *versus* Model, M_DEX0.05 *versus* Model, M_Escin *versus* Model or M_Escin_DEX0.05 *versus* Model. **(B)** Principal component analysis of the PCR array results. The colors of sample points are consistent with the SampleType of **(A)**. **(C)** The relative mRNA expression values of four key genes *Tnf*, *Il6*, *Nr3c1*, and *Sphk1*. Model: AIA; M_DEX0.2: AIA + Dex (0.2); M_DEX0.05: AIA + Dex (0.05); M_Escin: AIA + Escin; M_Escin_DEX0.05: AIA + Escin + Dex (0.05).

Considering that Dex specifically targeting GR, gene chips were further used to explore the combination mechanism between Escin and low GC (Dex) doses (Zhou et al., 2015). As illustrated in Figure 5, heat map and principal component analysis results of the gene chip revealed that the AIA group, AIA + Dex (0.05) group, and AIA + Escin group clustered together, which suggested that the AIA + Dex (0.05) group and AIA + Escin group had little influence on the GR pathway that was altered by AIA. AIA + Dex (0.2) clustered together with the control group, which suggested a significant anti-RA effect of Dex (0.2) in AIA-induced rats. However, AIA + Escin + Dex (0.05) group clustered together with the control group and AIA + Dex (0.2) group, which suggested that Escin combined with Dex (0.05) had a similar influence compared with Dex (0.2). Further analysis of specific genes showed that the control group, AIA + Dex (0.2) group and AIA + Escin + Dex (0.05) group shared the same trend in the *Tnf*, *Il-6*, *Nr3c1*, *Sphk1* mRNA expression. Moreover, these three groups improved the expression of *Nr3c1 (GR)* and the key genes, *Sphk1*, in the GR pathway, which suggested that Escin combined with Dex (0.05) might treat the disease by increasing GR expression and further affecting its relevant pathways.

### Confirmation of the GeneChip Results in AIA Rats

**FIGURE 6:**
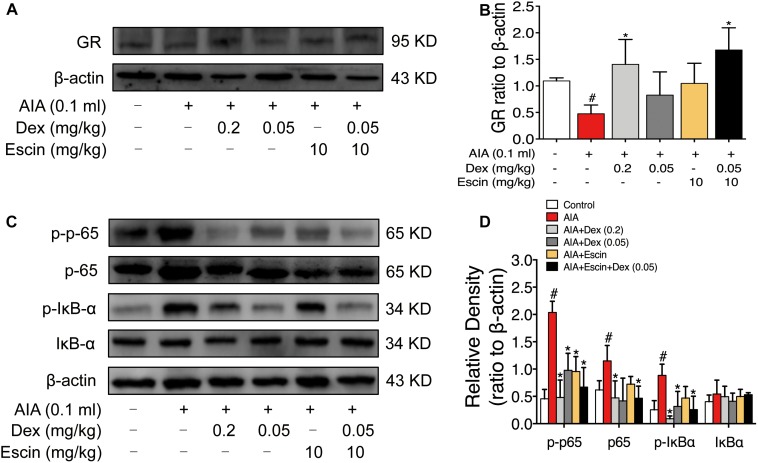
Escin combined with Dex activated GR in AIA rats. **(A,C)** Representative western blots of GR, *p*-p65, p65, *p*-IκBα, IκBα, and β-actin. **(B,D)** The quantified densitometric analysis of GR, *p*-p65, p65, *p*-IκBα, IκBα. Data were expressed as the mean ± SD, and were analyzed by ANOVA. ^#^*P* < 0.05 *versus* the control group; ^∗^*P* < 0.05 *versus* the AIA-treated group; Dex, dexamethasone.

As illustrated in Figure 6, GR expression was significantly down-regulated in the AIA-treated group (*P* < 0.05), and its expression was significantly up-regulated after Dex (0.2 mg/kg) and Escin (10 mg/kg) combined with Dex (0.05 mg/kg) administration (*P* < 0.05). Moreover, the AIA-treated group significantly increased *p*-p65, p65 and *p*-IκBα expression. When administrated with Dex (0.2 mg/kg), Escin (10 mg/kg) combined with Dex (0.05 mg/kg) significantly decreased *p*-p65, p65 and *p*-IκBα expression (*P* < 0.05). Dex (0.05 mg/kg) significantly decreased *p*-p65 and *p*-IκBα expression (*P* < 0.05). Escin (10 m/kg) significantly decreased *p*-p65 expression (*P* < 0.05).

### Confirmation of the Gene Chip Results in LPS-Induced RAW 264.7 Cells

**FIGURE 7:**
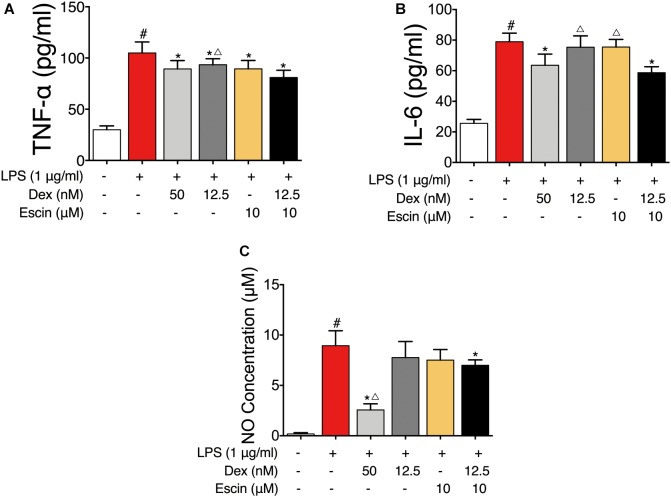
Anti-inflammation effect of Escin combined with Dex in LPS-induced RAW 264.7 cells. **(A,B)** Cell supernatant IL-6 and TNF-α were determined with ELISA kits. **(C)** Cell supernatant NO concentration was determined with Griess reagent kit. Data were expressed as the mean ± SD, and were analyzed by ANOVA. ^#^*P* < 0.05 *versus* the control group; ^∗^*P* < 0.05 *versus* the AIA-treated group; ^△^*P* < 0.05 *versus* the Escin (10 mg/kg) combined with Dex (0.05 mg/kg); Dex, dexamethasone.

To further confirm the gene chip results, the LPS-induced RAW264.7 cell model was constructed. As displayed in Figure 7, the TNF-α, IL-6 and NO concentrations were significantly increased in the LPS group (*P* < 0.05). Dex (50 nM) and Escin (10 μM) combined with Dex (12.5 nM) significantly decreased the TNF-α, IL-6 and NO concentrations (*P* < 0.05). Moreover, the TNF-α concentration in Escin (10 μM) combined with Dex (12.5 nM) group was significantly lower than the Dex (12.5 nM) group (*P* < 0.05). The IL-6 concentration in the Escin (10 μM) combined with Dex (12.5 nM) group was significantly lower than that in the Dex (12.5 nM) and Escin (10 μM) (*P* < 0.05) group.

**FIGURE 8:**
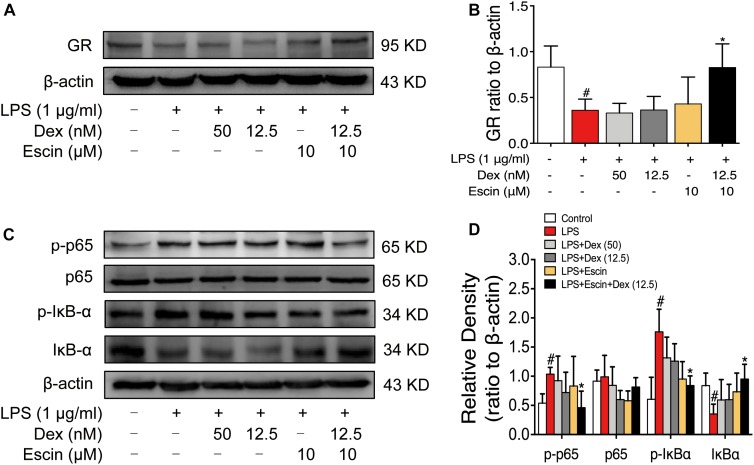
Escin combined with Dex activated GR in LPS-induced RAW 264.7 cells. **(A,C)** Representative western blots of GR, *p*-p65, p65, *p*-IκBα, IκBα and β-actin. **(B,D)** The quantified densitometric analysis of GR, *p*-p65, p65, *p*-IκBα, IκBα. Data were expressed as the mean ± SD, and were analyzed by ANOVA. ^#^*P* < 0.05 *versus* the control group; ^∗^*P* < 0.05 *versus* the AIA-treated group; Dex, dexamethasone.

We then confirmed GR activation in this cell model *in vitro*. The LPS-treated group significantly decreased GR expression (*P* < 0.05). Escin (10 μM) combined with Dex (12.5 nM) significantly increased GR expression (*P* < 0.05). Moreover, the LPS-treated group significantly increased *p*-p65, *p*-IκBα expression and decreased IκBα expression (*P* < 0.05). Administrated with Escin (10 μM) combined with Dex (12.5 nM) significantly decreased *p*-p65 and *p*-IκBα expression and increased IκBα expression (Figure 8) (*P* < 0.05).

### Escin Enhances the Effect of Low Dose Glucocorticoids Through GR Activation

**FIGURE 9:**
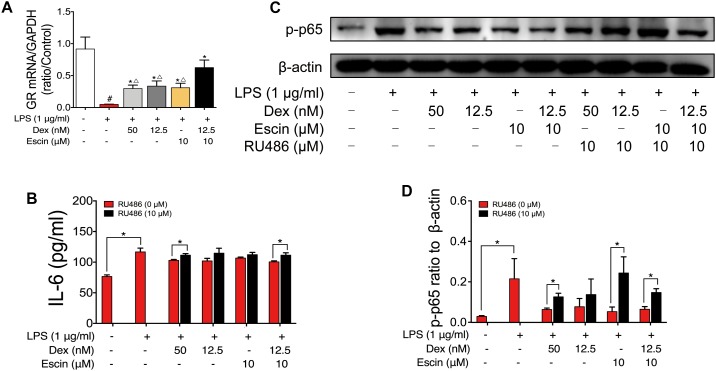
Escin enhances the effect of low dose glucocorticoids through GR activation. **(A)** GR mRNA expression was determined by RT-PCR assay. Data were expressed as the mean ± SD, and were analyzed by ANOVA. ^#^*P* < 0.05 *versus* the control group; ^∗^*P* < 0.05 *versus* the AIA-treated group; ^△^*P* < 0.05 *versus* the Escin (10 mg/kg) combined with Dex (0.05 mg/kg); Dex, dexamethasone. **(B)** Cell supernatant IL-6 was determined with ELISA kits. Data were expressed as the mean ± SD, and were analyzed by ANOVA. ^∗^*P* < 0.05; Dex, dexamethasone. **(C,D)** Expression of *p*-p65 was determined by western blotting. Data were expressed as the mean ± SD, and were analyzed by ANOVA. ^∗^*P* < 0.05; Dex, dexamethasone.

GR mRNA expression was also detected. As shown in Figure 9, LPS decreased GR mRNA expression significantly. Escin (10 μM) combined with Dex (12.5 nM) significantly increased the GR mRNA. When compared with Dex (50 nM), Dex (12.5 nM) and Escin (10 μM) groups, the Escin (10 μM) combined with Dex (12.5 nM) group displayed a higher GR mRNA expression (*P* < 0.05). Cell supernatant IL-6 concentration in the LPS treated group significantly increased (*P* < 0.05), and the IL-6 concentration in the Dex (50 nM), Dex (12.5 nM), Escin (10 μM), Escin (10 μM) combined with Dex (12.5 nM) groups was significantly decreased compared with LPS (*P* < 0.05). After pre-treatment with the GR specific inhibitor, RU486, the cell supernatant IL-6 concentration was significantly increased in the group treated with Dex (50 nM) and Escin (10 μM) combined with Dex (12.5 nM) (*P* < 0.05). In the LPS-treated group, *p*-p65 expression was increased significantly (*P* < 0.05). Dex (50 nM), Dex (12.5 nM), Escin (10 μM), and Escin (10 μM) combined with Dex (12.5 nM) significantly decreased *p*-p65 expression (*P* < 0.05). After pre-treatment with RU486, *p*-p65expression in Dex (50 nM), Escin (10 μM) and Escin (10 μM) combined with Dex (12.5 nM) group were increased.

## Discussion

Rheumatoid arthritis is a chronic systemic disease with clinical manifestations of multi-joints damage, leading to chronic pain, joint deformity and functional disability. There are many types of medications for RA, such as non-steroidal anti-inflammatory drugs (NSAIDs), disease-modifying anti-rheumatic drugs (DMARDs), biological agents and GCs (Scott et al., 2010). GCs are 21-carbon steroid hormones. They are still used in the treatment of RA although a variety of adverse effects exist. Because GCs show strong anti-inflammatory effects and can reduce signs and symptoms of the disease, exert disease-modifying effects, especially in the active stage of RA (Strehl et al., 2017). Indeed the adverse effects of GCs such as infections, immunosuppression and osteoporosis limited their widely clinic use (Dubois-Camacho et al., 2017). So some new treatment strategies for RA with GCs are under development. For example, previous studies reported that transactivation is responsible for most of the adverse reactions of GCs while transrepression is considered to mediate their anti-inflammatory effects (Barnes, 2017). Novel drugs such as selective GR agonists, also called dissociated agonists, are under development (Sundahl et al., 2015). Escin is a natural mixture of triterpene saponins isolated from *Aesculus hippocastanum* L. Based on the network pharmacology, the TCM network related to Escin was constructed. MM symptom muscle weakness was related to RA, and the TCM symptom such as Tong, Teng Tong was also closely related to RA. This was consistent with our previous study. Our previous studies found that Escin exerts synergistic anti-inflammatory effects with GCs (Xin et al., 2011b), shows anti-arthritic effects combined with low dose of GCs with reduced adverse effects (Du et al., 2016). The present study elucidated the possible anti-arthritic mechanism of Escin, which may be another strategy to improve the efficacy and diminish any possible adverse effects of GCs.

The rat AIA model is an easy, reliable, and reproducible experimental model of polyarthritis with a short duration. It has been commonly used for preclinical evaluation of anti-arthritic drugs because of its similar pathology of human RA (Wang et al., 2016). In the present study, we demonstrated that low-dose Dex combined with Escin exerts an enhanced protective effect in a rat model of AIA. After injection of adjuvant, clinical evidence of arthritis occurred and was gradually exacerbated in immunized rats. Escin and Dex were given orally. Rat paw swelling and serum inflammatory factors served as indicators of systemic inflammation. Results revealed that Escin (10 mg/kg) combined with Dex (0.05 mg/kg) and Dex (0.2 mg/kg) significantly decreased the arthritic index, reduced the paw swelling, decreased serum IL-6 and TNF-α levels, and ameliorated the joint pathology. The values of the indexes showed similar trends. However, Escin combined with Dex, improved the effect of the low dose Dex.

It is known to all that high dose and long-term use of glucocorticoids can lead to pleiotropic side effects, including immunosuppression. In the present study, we investigated the effect of Escin combined with Dex on immune organ histopathological change and index in AIA rats. The results demonstrate that Escin combined with low dose of Dex shows good anti-arthritic effects but not induces apoptosis of immune cells or cause damage to immune organs compared to high dose of Dex.

To explore the underlying combination mechanism, a GC signaling RT2 Profiler OCR Array was used. The results revealed that *Nr3c1*, *Asph*, *Got1*, *Il-6*, *Tnf*, and *Sphk1* were altered significantly, especially *Nr3c1*, which showed high expression in both Dex and Escin combined with Dex groups. This result suggests that activation of GR is key events in the combination mechanism between Escin and low doses Dex.

We further confirmed the activation of GR *in vivo* and *in vitro*. Our results revealed that Dex (0.2 mg/kg) and Escin (10 mg/kg) combined with Dex (0.05 mg/kg) significantly up-regulated GR expression in AIA-induced RA rats *in vivo*. The value of GR expression in Escin (10 mg/kg) combined with Dex (0.05 mg/kg) was higher than that of the Dex (0.2 mg/kg) alone. This result was further confirmed in the LPS-induced RAW 264.7 cells *in vitro*. Escin (10 μM) combined with Dex (12.5 nM) significantly up-regulated GR expression *in vitro*.

Ample amounts of evidence support the idea that GCs exert their beneficial effects, at least in part, via interference with the NF-κB signaling pathway (De Bosscher et al., 2006; Gossye et al., 2008). NF-κB signaling pathway activation was also confirmed in the present study. Dex (0.2 mg/kg) and Escin (10 mg/kg) combined with Dex (0.05 mg/kg) activated *p*-p65, p65 and *p*-IκBα expression in AIA rats *in vivo*, and they activated *p*-p65, *p*-IκBα and IκBα in LPS-induced RAW 264.7 cells *in vitro*. This suggests that GR activation plays an important role in the effects of the combination of Escin and Dex.

Additionally, we evaluated the GR mRNA expression. The results revealed that Escin (10 μM) combined with Dex (12.5 nM) showed a significant high GR mRNA expression, and the levels observed in the combination treatment group were higher than that any of drug used alone. These results suggested that activation of GR pathway might be the underlying anti-RA mechanism in the Escin (10 μM) combined with Dex (12.5 nM) group. Additionally, GR was suppressed by its specific inhibitor, RU486. After GR suppression, the cell supernatant IL-6 and the *p*-p65 expression levels were reversed, which further confirmed the important role of GR pathway underlying the effects in the Escin (10 μM) combined with Dex (12.5 nM) group.

## Conclusion

This study provided comprehensive evidence supporting the anti-RA effects of Escin combined with Dex and the underlying mechanisms of this combined medication. Escin combined with Dex reduced the dose of Dex, and exerts a significant anti-RA effects, which may also reduce the adverse effects of Dex. This combination might be attributed to GR activation. This study might provide a new combination of drugs for the treatment of RA.

## Author Contributions

LZ, YHu, CW, YD, MW, XW, YW, YHa, and TW were involved in data acquisition. FF, ZG, and BF conceived and designed the study. LZ, YHu, and PL have made statistical analyses. CW and LZ wrote the manuscript. All authors contributed to analysis and interpretation of the data and approved the final manuscript.

## Conflict of Interest Statement

The authors declare that the research was conducted in the absence of any commercial or financial relationships that could be construed as a potential conflict of interest.

## Footnotes

1 http://www.symmap.org
